# Chemoprophylaxis of *Dirofilaria immitis* (Leidy 1856) infection at a high challenge environment

**DOI:** 10.1186/s13071-015-1141-6

**Published:** 2015-10-12

**Authors:** Norma Vollmer Labarthe, Liliane Maria Valentim Willi, Jonimar Pereira Paiva, Marcia Gonçalves Nobre de Miranda, Karen Zoreck, Flavya Mendes de Almeida

**Affiliations:** Programa de Pós-Graduação em Medicina Veterinária, Faculdade de Veterinária, Universidade Federal Fluminense, Rua Vital Brazil 64, Santa Rosa, CEP 24230-340 Niterói, RJ Brazil; Programa Institucional Biodiversidade e Saúde, Fundação Oswaldo Cruz, Av. Brasil 4036, Manguinhos, CEP 21040-361 Rio de Janeiro, RJ Brazil; Doutoranda do Programa de Pós-Graduação em Medicina Veterinária, Faculdade de Medicina Veterinária, Universidade Federal Fluminense, Rua Vital Brazil 64, Santa Rosa, CEP 24230-340 Niterói, RJ Brazil; Departamento de Medicina e Cirurgia Veterinária, Instituto de Veterinária, Universidade Federal Rural do Rio de Janeiro, BR-465, Km 7, CEP 23890-000 Seropédica, RJ Brazil; Médica Veterinária Vet Ypiranga, Rua Ypiranga 107, Laranjeiras, CEP 22231-120 Rio de Janeiro, RJ Brazil; Bayer S.A., Bayer Technology Services, Av. das Américas, 500, Downtown Bloco 11, Loja 108, 26640-100 Rio de Janeiro, RJ Brazil

**Keywords:** Canine heartworm, Macrocyclic lactones, Preventive medication

## Abstract

**Background:**

The frequency of canine heartworm infection in the state of Rio de Janeiro, Brazil was high before chemoprophylactic treatment was available, with one of the highest rates of infection (52.5 %) found among dogs living on the eastern shore of the state. Following the launch of a chemoprophylactic product, the rate of infection gradually decreased, and new infections were rarely reported. After 2005, outbreaks reported at the eastern shore as well as for new infections in other areas of high infection frequency were considered to possibly be related to reduced efficacy of macrocyclic lactones. Therefore, this study aimed to evaluate the efficacy of topical heartworm preventatives from different drug families at the high challenge area of the state of Rio de Janeiro.

**Methods:**

A total of 46 dogs, including animals negative for *Dirofilaria immitis* microfilariae and antigen (Snap 4 Dx, IDEXX Laboratories, USA) at the initial screening were randomly allocated to two monthly treatment groups. Dogs in one group received topical moxidectin + imidacloprid and dogs in the other group received topical selamectin for eight consecutive months. Blood samples were obtained for microfilariae and antigen detection until the eleventh month after the first treatment. Dogs becoming microfilaremic or antigenemic on or before day 180 were considered to be infected prior to the first dose and were excluded from the study.

**Results:**

A total of 29 dogs completed the study, including 14 treated with moxidectin + imidacloprid and 15 treated with selamectin. No dogs treated with moxidectin + imidacloprid (0/14) became infected during the treatment period, whereas four dogs of the selamectin group (4/15) became infected.

**Conclusion:**

Topical moxidectin + imidacloprid is 100 % effective in preventing *D. immitis* infections in dogs living in a high challenge natural environment.

## Background

*Dirofilaria immitis* (Leidy, 1856) Raillet & Henry, 1911 is a mosquito-borne parasite species present worldwide and is frequently found infecting Brazilian dogs [1]. As in other areas of the world, its transmission depends on the presence of microfilaremic dogs, competent mosquito vectors, and susceptible dogs [2, 3].

Before the year of 1992, when the first chemoprophylactic treatment was launched in Brazil, reported canine heartworm infection frequency at the state of Rio de Janeiro ranged from 14 % to 17 % [4, 5]. During those years (1988–1990), at the eastern shore of the state, the frequency was as high as 52.5 % [5]. During the following years (2000–2001), after the first chemoprophylactic product was launched, a downward trend in heartworm infection occurred in Brazil [6, 7], and new infections were rarely reported.

In 2005, following the availability of chemoprophylactic heartworm treatments, the first heartworm outbreak in the state was identified in the area of the eastern shore, with 53 % of the dogs found to be infected [8]. Following the first outbreak, many other infections were reported, and lack of efficacy of macrocyclic lactones in preventing infections was suspected in high infection frequency areas of the state [9]. An update of the 2005 outbreak area six years later showed that infection frequency was even higher (80 %) [10, 11], which, along with local veterinarians reports that dogs on chemoprophylaxis were becoming infected (personal communication), suggests the presence of less susceptible populations of *D. immitis* at the site. Therefore, the aim of this study was to evaluate the efficacy of topical heartworm preventatives from different drug families at the high challenge area of the state of Rio de Janeiro.

## Methods

In order to identify as many as possible *D. immitis* microfilariae and antigen negative dogs living at the high challenge area, all dog owners were invited to participate of the study. According to the approval of the ethical committee of the Universidade Federal Fluminense (233) and after obtaining owner consent, blood samples were obtained from 80 chemoprophylaxis naïve dogs (32 under 12 months old; 19 from 1 to 2 years old and 29 over 2 years) for detection of microfilariae using Knott´s modified test (Newton & Wright, 1956) and ELISA test (Snap 4 Dx IDEXX Laboratories, USA).

**Fig. 1:**
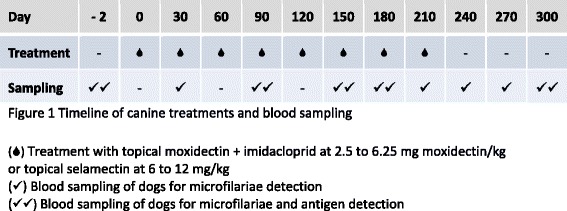
Timeline of canine treatments and blood sampling.

After screening, 46 out of the 49 dogs with negative test results on both tests (26/46, 56.5 % less than 1 year of age) were included in the study and randomly sorted in two groups; dogs in one group received topical moxidectin + imidacloprid at 2.5 to 6.25 mg moxidectin/kg/month (23 dogs); dogs in the second group received topical selamectin at 6 to 12 mg/kg/month (23 dogs). Every monthly treatment was administered by the veterinarians participating in the study after weighing the dogs and adjusting the dose if necessary. Dogs were treated on days 0, 30, 60, 90, 120, 150, 180 and 210 (±5 days), and blood samples were obtained on days −2, 30, 90, 150, 180, 210, 240, 270, and 300 (±5 days) for microfilariae detection. On days −2, 90, 150, 180, and 300, plasma samples were tested for detection of antigens by the same ELISA test used for the initial screening. Plasma from all microfilaremic samples obtained on days 30, 210, 240, or 270 was also tested for antigens (Fig. 1).

Dogs becoming microfilaremic or antigenemic on day 180 or before were considered to be infected prior to the first chemoprophylactic dose. Those dogs and any other inadvertently treated with macrocyclic lactones by the owners were excluded from the study.

Detection of infected dogs during the study were evaluated for significance of differences between the treatment groups by chi square or Fisher’s exact tests.

## Results

The initial screening of 80 dogs detected 31 positive for microfilariae and/or *D. immitis* antigen (38.8 %). Among the 46 dogs included, 14 of the moxidectin + imidacloprid group and 15 of the selamectin group completed the study. On day 180, one of the selamectin dogs became microfilaremic and became antigen positive later. Therefore, this dog was considered to have been previously infected before the study was initiated. The number of dogs in the selamectin group that became infected during the treatment period (4/15) was higher than the moxidectin + imidacloprid treated group (0/14) (*χ*^2^ = 2.38; p = 0.057) (Table 1). Among the four selamectin-treated dogs that became infected during the study, three tested positive for microfilariae and antigen and one was antigen-positive but was amicrofilaremic. Therefore the efficacy of the selamectin treatment was 73.3 % and the efficacy of the moxidectin + imidacloprid treatment was 100 %.

**Table 1:** Number and percentage of dogs receiving monthly treatment with topical moxidectin + imidacloprid or selamectin treatment positive for microfilariae or antigen of *Dirofilaria immitis* at different study intervals from day 30 (after first treatment) up to day 300 after the first treatment

	Day 30 to 150^a^	Day 180^b^	Day 270 to 300
Monthly treatment	No. pos./total	No. pos./total	No. pos./total
Moxidectin + imidacloprid	3/23 (13.0 %)	0/18 (0 %)	0/14^a^ (0 %)
Selamectin	6/23 (26.1 %)	1/20 (5.0 %)	4/15^b^ (26.7 %)

^a^Considered to be infected before the first treatment

^b^Possibly infected before the first treatment

Different superscript letters in columns are significantly different (*x* ^2^ = 2.38; *p* = 0.057)

## Discussion

Because dogs had to be free of detectable microfilariae and antigens to be included in the study and the challenge at the study site was known as very high, young dogs were privileged. Even so, the percent of positive test results was high (38.75 %), confirming previous results [11] and demonstrating how intense the challenge was. Therefore, the severe life threatening cardiopulmonary condition the infection determines, the risk treatment imposes to the dogs and the zoonotic potential of the infection highlights the definite need for chemoprophylaxis in order to control transmission. Minimizing the number of infected dogs acting as reservoirs of the parasite is pivotal to reduce the chance of infective blood meals for mosquitoes and to reduce the occurrence of *D. immitis* in vectors, dogs and humans. Besides the direct impact upon *D. immitis* transmission, the use of topical moxidectin/imidacloprid or topical selamectin provides reliable control against other major endo and ectoparasites.

The main reasons for macrocyclic lactones treatment failures which may impair analysis of lack of efficacy or resistance are known to be underdosing and owners’ noncompliance [12–15], which were carefully controlled in the present study. Therefore, the results obtained ruled out major indirect reasons for lack of efficacy at a high natural challenge area were the occurrence of resistant populations or lack of efficacy can´t be disregarded.

Topical moxidectin + imidacloprid was more efficacious (100 %) than topical selamectin (73.3 %) in preventing *D. immitis* infection, as it had been shown before when the resistant strains MP3 or JYD-34 were used as models for evaluation of efficacy [13, 16, 17]. Furthermore, it should be considered that since macrocyclic lactones may stunt *D. immitis* development for nine months when administered accordingly to recommendations (John Wilson McCall - personal communication), it is possible to assume that infection of the selamectin dogs occurred prior to the first treatment dose and that selamectin treatment was ineffective in eliminating the infection although stunted worm development.

Therefore, it is possible to infer that topical moxidectin + imidacloprid, when consistently administered to dogs on a monthly schedule, is highly effective in preventing heartworm patent infections in treated dogs living in extreme challenge situations, such as those found in the coastal lowland of the eastern state of Rio de Janeiro, Brazil [8, 9, 11, 18], the Mississippi delta in the United States [12], and others.

## Conclusions

Topical moxidectin + imidacloprid was 100 % effective for *D. immitis* prevention in dogs living in a high challenge natural environment therefore at those environments should be the chemoprophylaxis of choice. These results suggest that experimental studies should be conducted to elucidate the stunting effect of different macrocyclic lactones consistent treatment.
